# Liquid-Liquid Equilibrium of Poly(Ethylene Glycol) 6000 + Sodium Succinate + Water System at Different Temperatures

**DOI:** 10.1155/2013/819259

**Published:** 2013-06-20

**Authors:** Selvaraj Raja, Vytla Ramachandra Murty

**Affiliations:** Department of Biotechnology, Manipal Institute of Technology, Manipal, Karnataka 576104, India

## Abstract

Phase diagrams and the compositions of coexisting phases of poly(ethylene glycol) (PEG) 6000 + sodium succinate + water system have been determined experimentally at 298.15, 308.15, and 318.15 K. The effects of temperature on the binodal curve and tie lines have been studied. The binodal curves were successfully fitted to a nonlinear equation relating the concentrations of PEG 6000 and sodium succinate, and the coefficients were estimated for the formentioned systems (low AARD, high *R* ^2^, and low SD). Tie-line compositions were estimated and correlated using Othmer-Tobias and Bancroft equations, and the parameters were reported. The effect of temperature on the phase-forming ability has been studied by fitting the binodal data to a Setschenow-type equation for each temperature. The effective excluded volume (EEV) values were also calculated from the binodal data, and it was found out that the values increased with an increase in the temperature. Furthermore, the effect of MW of PEG on the phase diagram has been studied and verified.

## 1. Introduction

A liquid-liquid extraction method, Aqueous Two-Phase System (ATPS), comprised of a hydrophilic polymer and salt with water (polymer + salt + water) has been proved as a frugal and powerful method to separate and partially purify various biomolecules from the crude fermentation broth as compared to ATPS consists of two hydrophilic polymers with water (polymer + polymer + water) [1]. It has been shown by many researchers that this downstream processing method reduces the number of initial purification steps and thus decreases the overall processing cost [2–4]. Apart from conventional purification of biomolecules, ATPS also finds its applications in environmental remediation such as removal of color from textile effluents [5], metal removal from effluents [6], and recovery of biomolecules from various industrial effluents [7–10]. In our lab, we have established a citrate-based ATPS to recover valuable biomolecules from tannery wastewater [11, 12]. 

The type of the phase components used affects the properties of an ATPS. Because of the salient features like low cost, ease of phase separation with salts, and enhancement of the refolding of proteins to recover the activity, poly(ethylene glycol) (PEG) is the most commonly used polymer in ATPS [13]. Now there is a considerable interest in using biodegradable salts with PEG to formulate ATPS instead of conventional inorganic salts like phosphates and sulfates. The presence of inorganic salts in the effluent imposes environmental pollution, and it can be avoided by using biodegradable salts like citrates, tartrates, and succinates for the formulation of ATPS [14]. 

Even though there is a significant research work done on PEG + citrate + water system, ATPS based on PEG + succinate + water system is very limited and is not explored much. According to our knowledge there are only three [15–17] reports available for PEG + succinate + water system. Ananthapadmanabhan and Goddard [15] reported binodal data for PEG 6000 + succinate + water at room temperature; Zafarani-Moattar and Hamzehzadeh [16] reported the binodal data and tie-line compositions of PEG 6000 + succinate + water at 298.15 K; Perez et al. [17] studied the binodal behavior of PEG 6000 + succinate + water at 295.1 K and partitioning of various standard proteins. However, the data for other temperatures for these systems are not available in the literature.

Development of any ATPS involves the phase diagram determination which gives the information about the concentration of phase forming components necessary to form two phases and the phase composition after the phase separation. This data is very much essential to design an ATPS for the separation and purification of biomolecules which may be used for single-stage extraction or multistage extraction processes [18]. 

Therefore the aim of the present study is to develop the unexplored, liquid-liquid equilibrium (LLE) data of PEG 6000 + sodium succinate + water at different temperatures 298.15 K, 308.15 K, and 318.15 K and tie-line compositions. The effect of temperature in the present system was discussed on the basis of salting-out coefficient and effective excluded volume and compared with the literature. The effect of molecular weight of PEG was also discussed.

## 2. Materials and Methods

### 2.1. Materials

PEG 6000 and PEG 10000 from Merck and sodium succinate heptahydrate from Loba chemicals, India were used for the present work. All the chemicals were used without further purification and Millipore-Milli-Q water was used in all the experiments. 

### 2.2. Construction of Binodal Curve

Titration method [19] was used to obtain binodal curves. The experiments were carried out in a 100 mL jacketed glass vessel. The temperature of the working vessel was maintained at various temperatures with an uncertainty of ±0.1 K (Refrigerated Circulating Bath, EQUIBATH, no. 85006) by circulating water through an external jacket. Aqueous stock solution of PEG 6000 of 40% (w/w) and sodium succinate of 30% (w/w) were prepared and kept in a constant-temperature bath. After equilibration, a known amount of PEG stock solution was taken in the jacketed vessel and constantly stirred by a magnetic stirrer in order to maintain uniform concentration of the components of ATPS. A known amount of the salt solution was added drop-wise to the PEG solution or vice versa, in the jacketed vessel till the appearance of turbidity, which indicated the two-phase formation. To this two-phase solution, water was then added drop-wise until the disappearance of turbidity (single phase). The procedure was repeated to get enough binodal points. An analytical balance with a precision of ±0.1 mg (Shimadzu Analytical Balance, Japan, Model: AUW-120D) was used to determine the composition of the mixture. All the experiments were done in duplicates and average values were reported. 

### 2.3. Determination of Tie-Line Length and Composition

For the determination of tie-line composition, a series of ATPSs of known total compositions were prepared in graduated 15 mL centrifuge tubes and placed in the thermostatic water bath at various temperatures. The solution was rigorously mixed in a vortex mixer for 10 min. This mixture was equilibrated for 24 h in the thermostatic water bath at the specified temperatures. The two individual phases were carefully separated and the concentration of sodium ions in the top and bottom phases was determined by flame photometry (Systronics128 flame photometer). The uncertainty of the mass fraction of the salt was within ±0.002. The equilibrium concentration of PEG in both phases was determined by refractive index measurements using an Abbe-type refractometer (Advance Research Instruments Co., New Delhi, Model R-4) with a precision of ±0.0001. Samples were properly diluted in such a way that the concentration would fall within the linear range of calibration. 

## 3. Results and Discussions

### 3.1. Binodal Curve

**Figure 1:**
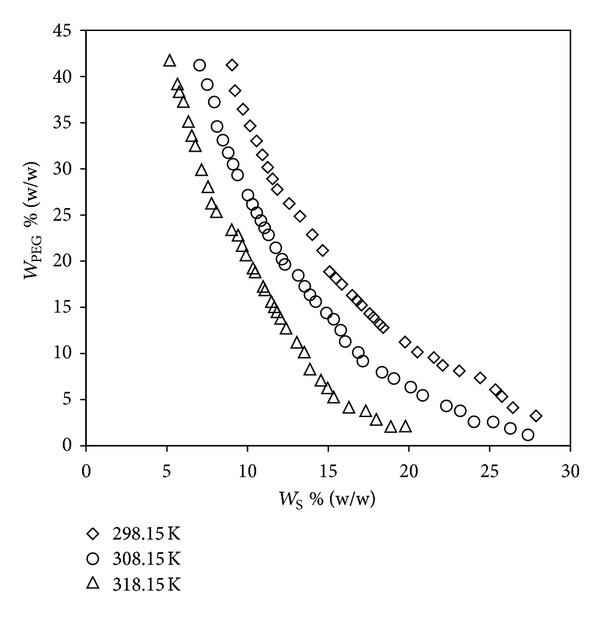
Effect of the temperature on the location of binodal curves for the PEG 6000 (*W*_PEG_) + sodium succinate (*W*_S_) + water systems.

The region below the binodal curve represents single-phase and above it represents two-phase region, and thus a binodal curve is a boundary line between them. The binodal data obtained by the titration method for PEG 6000 + sodium succinate + water systems at various temperatures are given in Table 1 and are shown schematically in Figure 1.

There are several correlations available in the literature to correlate with the binodal data [20–22]. 

However, the nonlinear equation (1) proposed by Hu et al. [23] was best fitted for the present experimental data. Consider

(1) $$\begin{array}{c} W_{\text{PEG}}=A+BW_{\text{S}}^{0.5}+CW_{\text{S}}+DW_{\text{S}}^{2\;}, \end{array}$$

where *W*_PEG_ and *W*_S_ are the weight percentages of PEG and sodium succinate, respectively. *A*, *B*, *C*, and *D* are the fitted parameter coefficients. The fitted coefficients are shown in Table 2. The high regression coefficient (*R* ^2^) values, less average arithmetic relative deviation (AARD), and standard deviation (SD) values suggest that the experimental data are well fitted with the correlations.

Figure 1 reveals that there is an expansion of biphasic area as the temperature increases and it moves towards the origin. This behavior is due to the increase in salt solubility and the concentration of salt and PEG required for the formation ATPS decreases with increasing the temperature. Similar results were reported by many researchers [24, 25] for other ATPS at different temperatures. This kind of binodal behavior can be explained on the basis of effective excluded volume (EEV). It is the available space in a network of one component (PEG) to occupy the other component (salt); it also characterizes the acceptability of a particular salt by a polymer to form an ATPS, with water as a solvent. This value is affected by the size, shape, and molecular interactions of the phase components involved in ATPS [26].

In the present work, the model (2) proposed by Guan et al. [26] which was based on statistical geometry was used to determine the EEV values. Consider

(2) $$\begin{array}{c} \ln\left[V_{123}^{*}\frac{W_{\text{PEG}}}{M_{\text{PEG}}}\right]+\left[V_{123}^{*}\frac{W_{\text{S}}}{M_{\text{S}}}\right]=0, \end{array}$$

where *V*_123_* is the effective excluded volume and *M*_PEG_ and *M*_S_ are the molecular weights of PEG and the sodium succinate, respectively. The EEV values were calculated by regression analysis of the model (2) and given in Table 3.

In the investigated systems it has been observed from Table 3 that EEV is relatively more at higher temperatures, indicating that salting-out strength of the salt increases with an increase in temperature. This high salting-out ability at high temperature is reflected in Figure 1 by a positional shift of binodal curve towards the left side, and thus a less concentration of salt is needed to form two-phase system. This is in accordance with the reported literature for other systems [24, 25]. 

### 3.2. Effect of Anion

**Figure 2:**
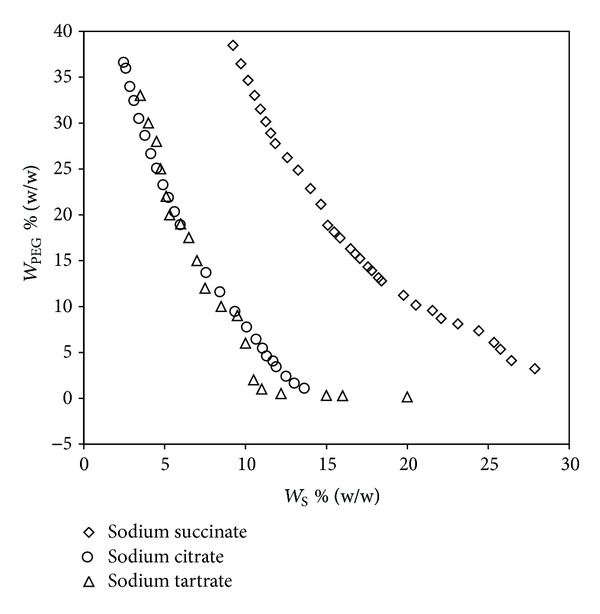
Effect of the cation on the binodal curves for the PEG 6000 (*W*_PEG_) + salt (*W*_S_) + water systems at 298.15 K.

In order to understand the effect of anion, the binodal curves of various salts having different anion and common sodium cation (sodium citrate, sodium tartrate, and sodium succinate) were plotted in Figure 2. The EEV values were obtained (Table 4) by calculation using the literature and experimental data. From the table it is clear that the EEV values follow the order citrate > tartrate > succinate, and this reveals that citrate anion has the higher valency than others and has the highest salting-out ability. Similar results were observed by Perez et al. [17]. Moreover this is in agreement with the familiar Hofmeister series [27] for anions which follows the order: citrate > tartrate > succinate. According to this series, the ions present in the left of the series increase the solvent surface tension and decrease the solubility of nonpolar molecules and thus have high salting-out ability. In addition to this, anions with higher valence are better salting-out agents because of their higher degree of hydration.

### 3.3. Tie-Line Compositions and Correlations

**Figure 3:**
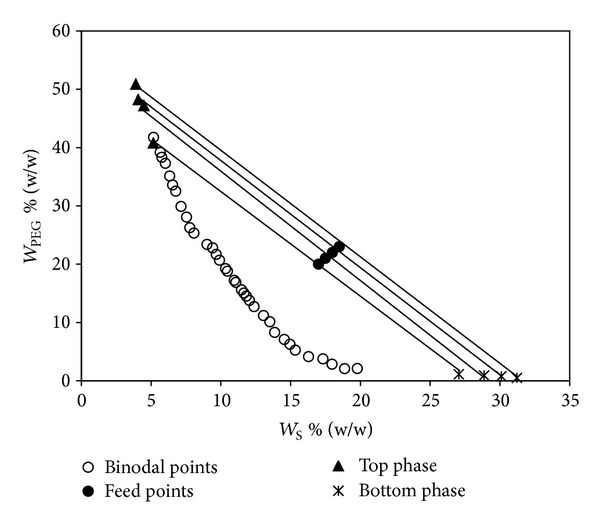
Binodal curve and tie lines for PEG 6000 (*W*_PEG_) + sodium succinate (*W*_S_) + water system at 318.15 K.

The equilibrium phase compositions of the two phases were related by tie-line length (TLL) which is defined as the difference in concentration of phase forming components of ATPS. It can be determined by the following equation and is expressed in weight percentage (% w/w):

(3) $$\begin{array}{c} \text{TLL}=\sqrt{{\left[W_{\text{S}}^{\text{T}}-W_{\text{S}}^{\text{B}}\right]}^{2}+{\left[W_{\text{PEG}}^{\text{T}}-W_{\text{PEG}}^{\text{B}}\right]}^{2}}, \end{array}$$

where *W*_S_ and *W*_PEG_ are the concentrations of salt and PEG, respectively, and superscripts T and B represent top and bottom phases, respectively. A series of TLLs in the two-phase region of the binodal curve were investigated and given in Table 5 and one set of data for 318.15 K is shown in Figure 3.

It is apparent from Table 5 that the increase in TLL increases the concentration of salt in the bottom phase and decreases the PEG concentration and vice versa. This is because of the increase in hydrophobicity of the phases as the TLL increases. Moreover, for the same feed compositions, the TLL increases as the temperature increases and it may be due to the increase in PEG hydrophobicity with an increase in temperature and the water is moved out from the polymer-rich top phase to the salt-rich bottom phase [28]. For all the systems investigated, the total system composition has no significant effect upon the slope of the tie lines, which implies that they are parallel to each other, thus allowing us to know the coexisting phase compositions for any given total polymer phase-forming composition [29].

The tie-line compositions of the PEG 6000 + sodium succinate + water fitted with Othmer-Tobias equation (4) and Bancroft equation (5) [30, 31]

(4) $$\begin{array}{c} \left(\frac{1-W_{\text{PEG}}^{t}}{W_{\text{PEG}}^{t}}\right)=K_{\text{OT}}{\left(\frac{1-W_{\text{S}}^{b}}{W_{\text{S}}^{b}}\right)}^{n}, \end{array}$$

(5) $$\begin{array}{c} \left(\frac{W_{\text{W}}^{b}}{W_{\text{S}}^{b}}\right)=K_{B}{\left(\frac{W_{\text{W}}^{t}}{W_{\text{PEG}}^{t}}\right)}^{r}, \end{array}$$

where *W*_W_ is the weight fraction of water and *K*_OT_, *n*, *K*_*B*_, and *r* are the fitted parameter coefficients. For the present system, these values are calculated and given in the Table 6.

**Figure 4:**
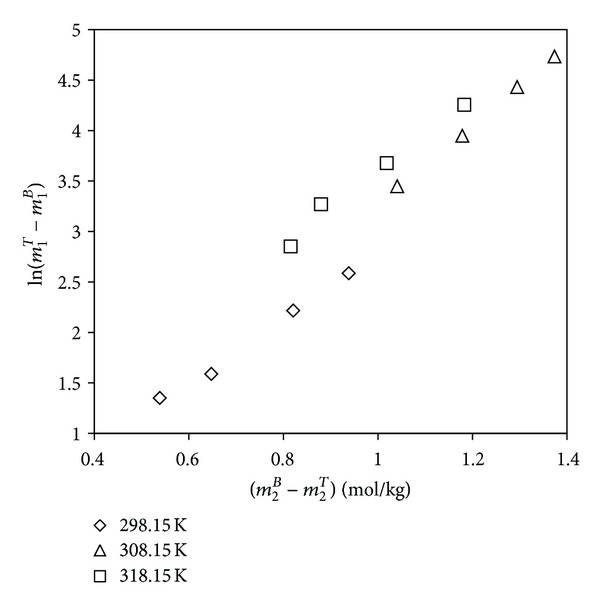
Setschenow-type plots for the type of salt on binodal curves for the PEG 6000 + sodium succinate + water systems for various temperatures.

The increase in hydrophobicity of PEG with increase in temperature can be related to the salting-out effect and is usually related to the empirical equation of modified Setschenow equation [32]. The equation used has the following form:

(6) $$\begin{array}{c} \ln\left(\frac{m_{\text{PEG}}^{\text{T}}}{m_{\text{PEG}}^{\text{B}}}\right)=K_{\text{PEG}}\left(m_{\text{PEG}}^{\text{B}}-m_{\text{PEG}}^{\text{T}}\right)+K_{s}\left(m_{\text{S}}^{\text{B}}-m_{\text{S}}^{\text{T}}\right), \end{array}$$

where *K*_PEG_ and *K*_*s*_ are the parameters relating the activity coefficient of PEG to its concentration and the salting-out coefficient, respectively. “*m*” represents molality and the superscripts T and B represent top phase and bottom phase, respectively. The parameters of the previous equations were calculated based on the tie-line data and shown in Table 7 and Figure 4.

It has been observed that the salting-out coefficient *K*_*s*_ value increases with increase in temperature. This is in accordance with the salting-out ability with EEV values as described earlier. Therefore two-phase formation occurs at lower concentration of phase components at higher temperatures. 

### 3.4. Effect of MW of PEG

**Figure 5:**
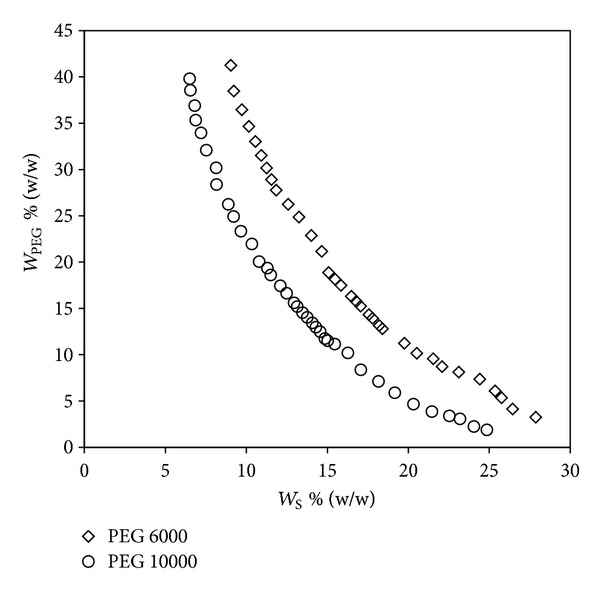
Effect of molecular weight of PEG on the binodal curve for the PEG (*W*_PEG_) + sodium succinate (*W*_S_) + water systems at 298.15 K.

In order to visualize the effect of PEG molecular weight, experiments were performed at 298.15 K for PEG 10000 + SS + water and compared with PEG 6000 + SS + water system (Figure 5). It is evident from the figure that as the MW increases the biphasic region expands and moves towards the origin thus requiring a lower concentration for phase separation. This behavior may be due to the high hydrophobicity of PEG 10000 when compared to PEG 6000 and thus a decrease in compatibility between phase components [22]. Moreover the EEV value for the present PEG 10000 + sodium succinate + water system was calculated by the procedure discussed earlier and it was found to be 53.62 g/mol which was lesser than PEG 6000 + sodium succinate + water system (37.62 g/mol). This confirms the higher salting-out characteristics of higher MW PEG [9].

## 4. Conclusions

The binodal curves for PEG 6000 + sodium succinate + water system at various temperatures (298.15 K, 308.15 K, and 318.15 K) were constructed and satisfactorily fitted with a non-linear equation. Increase in temperature resulted in the expansion of the binodal area and increase in effective excluded volume values because of high salting-out ability at high temperatures. The effect of anion on the location of binodal curve was also discussed and compared with the literature which followed the Hofmeister series: citrate > tartrate > succinate. The tie-line compositions were correlated with Othmer and Tobias equations and salting-out ability at various temperatures were fitted by Setschenow-type equation. The salting-out coefficient values increased with an increase in temperature similar to EEV values. The effect of PEG 10000 on the location of binodal curve was also verified. 

## Figures and Tables

**Table 1:** Binodal data for the PEG 6000 + sodium succinate + water system at different temperatures.

298.15 K		308.15 K		318.15 K	
*W*_PEG_ % (w/w)	*W*_*S*_ % (w/w)	*W*_PEG_ % (w/w)	*W*_*S*_ % (w/w)	*W*_PEG_ % (w/w)	*W*_*S*_ % (w/w)
41.23	9.05	39.12	7.51	41.74	5.18
38.46	9.23	37.23	7.94	39.19	5.67
36.45	9.72	34.58	8.12	38.33	5.77
34.64	10.16	33.10	8.47	37.28	6.03
33.00	10.56	31.74	8.80	35.12	6.34
31.51	10.92	30.49	9.11	33.59	6.55
30.15	11.26	29.33	9.39	32.51	6.77
28.90	11.56	27.13	10.03	29.89	7.15
27.75	11.84	26.15	10.31	28.06	7.55
26.23	12.59	25.24	10.58	26.25	7.78
24.86	13.26	24.39	10.82	25.33	8.08
22.85	14.02	23.60	11.05	23.39	9.02
21.14	14.66	22.83	11.30	22.79	9.42
18.86	15.09	21.43	11.75	21.67	9.68
18.14	15.48	20.20	12.14	20.66	9.91
17.47	15.84	19.64	12.32	19.24	10.34
16.28	16.50	18.44	13.15	18.80	10.48
15.74	16.79	17.27	13.54	17.24	10.97
14.32	17.57	16.36	13.87	16.88	11.08
13.90	17.80	15.62	14.22	15.61	11.49
13.14	18.22	14.38	14.89	15.04	11.66
12.78	18.41	13.71	15.34	14.51	11.83
11.22	19.77	12.52	15.77	13.79	12.06
10.15	20.53	11.29	16.06	12.73	12.39
9.55	21.55	10.09	16.87	11.19	13.06
8.69	22.09	9.17	17.14	10.13	13.52
8.11	23.12	7.96	18.33	8.29	13.87
7.35	24.42	6.35	20.11	7.09	14.55
5.33	25.76	5.46	20.86	5.27	15.34
4.11	26.44	4.31	22.32	4.16	16.29
3.22	27.88	3.79	23.18	3.78	17.33
		2.59	24.03	2.85	17.98
		2.57	25.22	2.13	19.79
		1.87	26.29	2.09	18.88
		1.18	27.38		

**Table 2:** Binodal curve coefficients of (1) for PEG 6000 + sodium succinate + water at various temperatures.

Temperature, K	*A*	*B*	*C*	*D*	*R* ^2^	AARD* (%)	SD**
298.15	247.48	−111.43	14.98	−0.09	0.9823	4.07	0.55
308.15	178.92	−74.87	8.92	−0.04	0.9886	8.10	0.52
318.15	103.12	−25.96	−0.97	0.08	0.9958	5.38	0.76

*Average arithmetic relative deviation (AARD) = {∑_*i*_^*N*^(||Expt − Cal/Expt||)/*N*} × 100.

**Standard deviation (SD) = {∑_*i*_^*N*^(Expt−Cal)^2^/*N*}^1/2^.

**Table 3:** EEV values (2) of PEG 6000 + sodium succinate + water at different temperatures.

Temperature, K	EEV (g/mol)	AARD, %	SD
298.15	37.62	2.70	1.28
308.15	43.18	2.18	1.07
318.15	50.89	3.46	2.13

**Table 4:** EEV values (2) of PEG 6000 + sodium succinate + water at 298.15 K.

Type of salt	EEV (g/mol)	SD	Reference
Sodium citrate	70.79	4.60	[33]
Sodium tartrate	61.59	7.94	[34]
Sodium succinate	39.32	2.35	[16]
Sodium succinate	37.62	1.28	This work

**Table 5:** Tie-line composition for the PEG 6000 + sodium succinate + water system at different temperatures.

TLL (% w/w)	Feed		Top phase		Bottom phase	
	*W*_PEG_ (% w/w)	*W*_*S*_ (% w/w)	*W*_PEG_ (% w/w)	*W*_*S*_ (% w/w)	*W*_PEG_ (% w/w)	*W*_*S*_ (% w/w)
298.15 K						
25.28	20.0	17.0	30.04	11.21	7.79	23.21
30.93	21.0	17.5	34.45	10.13	7.05	24.49
38.51	22.0	18.0	38.21	9.08	4.17	27.09
43.60	23.0	18.5	41.68	8.79	3.14	29.17
308.15 K						
39.55	20.0	17.0	37.24	7.98	2.15	26.22
42.43	21.0	17.5	39.15	7.53	1.49	27.07
46.28	22.0	18.0	41.87	6.51	1.06	28.34
50.75	23.0	18.5	45.09	6.13	0.64	30.61
318.15 K						
45.29	20.0	17.0	40.78	5.17	1.13	27.06
52.29	21.0	17.5	47.16	4.49	0.91	28.88
54.10	22.0	18.0	48.18	4.09	0.75	30.11
57.28	23.0	18.5	50.85	3.91	0.51	31.23

**Table 6:** Values of parameters of Othmer-Tobias and Bancroft equations PEG 6000 + sodium succinate + water system for various temperatures.

Temperature, K	*K*_OT_	*n*	*R* ^2^	*K*_*B*_	*r*	*R* ^2^
298.15	0.346387	1.5587	0.9687	2.150499	0.5044	0.9762
308.15	0.354623	1.4939	0.9875	2.153081	0.6407	0.9855
318.15	0.205913	1.9427	0.9507	1.615751	0.8477	0.9469

**Table 7:** Values of *K*_*s*_ and intercept of Setschenow-type equation (6) for the PEG 6000 + sodium succinate + water systems at various temperatures.

Temperature, K	*K*_*s*_ (kg/mol)	Intercept	*R* ^2^
298.15	3.1809	−0.4084	0.9934
308.15	3.6436	−0.0375	0.984
318.15	3.887	−0.6106	0.9565
